# Circulating Lipoprotein Sphingolipids in Chronic Kidney Disease with and without Diabetes

**DOI:** 10.3390/biomedicines12010190

**Published:** 2024-01-15

**Authors:** Maria F. Lopes-Virella, Samar M. Hammad, Nathaniel L. Baker, Richard L. Klein, Kelly J. Hunt

**Affiliations:** 1 Department of Medicine, Division of Diabetes, Endocrinology and Medical Genetics, Medical University of South Carolina, Charleston, SC 29425, USA; 2 Ralph H. Johnson VA Medical Center, Charleston, SC 29401, USA; huntke@musc.edu; 3 Department of Regenerative Medicine and Cell Biology, Medical University of South Carolina, Charleston, SC 29425, USA; 4 Department of Public Health Sciences, Medical University of South Carolina, Charleston, SC 29425, USA; bakern@musc.edu

**Keywords:** diabetes, macroalbuminuria, albuminuria, sphingolipid, sphingomyelin, glycosphingolipid, ceramide, hexosylceramide, lactosylceramide, sphingosine, dihydrosphingosine

## Abstract

Abnormalities of sphingolipid metabolism play an important role in diabetes. We compared sphingolipid levels in plasma and in isolated lipoproteins between healthy control subjects and two groups of patients, one with chronic kidney disease without diabetes (ND-CKD), and the other with type 2 diabetes and macroalbuminuria (D-MA). Ceramides, sphingomyelins, and sphingoid bases and their phosphates in LDL were higher in ND-CKD and in D-MA patients compared to controls. However, ceramides and sphingoid bases in HDL2 and HDL3 were lower in ND-CKD and in D-MA patients than in controls. Sphingomyelins in HDL2 and HDL3 were lower in D-MA patients than in controls but were normal in ND-CKD patients. Compared to controls, lactosylceramides in LDL and VLDL were higher in ND-CKD patients but not in D-MA patients. However, lactosylceramides in HDL2 and HDL3 were lower in both ND-CKD and D-MA patients than in controls. Plasma hexosylceramides in ND-CKD patients were increased and sphingoid bases decreased in both ND-CKD and D-MA patients. However, hexosylceramides in LDL, HDL2, and HDL3 were higher in ND-CKD patients than in controls. In D-MA patients, only C16:0 hexosylceramide in LDL was higher than in controls. The data suggest that sphingolipid measurement in lipoproteins, rather than in whole plasma, is crucial to decipher the role of sphingolipids in kidney disease.

## 1. Introduction

Sphingolipids are a class of lipids that comprise basic components of cell membranes and involve signaling molecules that regulate several vital cellular functions [1,2,3,4]. Glycosphingolipids are sphingolipids, which are particularly abundant in renal cells including podocytes, mesangial cells, and tubular epithelial cells, and were shown to play a critical role in kidney metabolism [5].

Chronic kidney disease (CKD) is characterized by “the presence of kidney damage or reduction of the glomerular filtration rate (GFR) for more than three months and it is classified into stages based on GFR values” [6]. A wide range of risk factors, including diabetes, hypertension, oxidative stress, and inflammation can contribute to the onset of CKD. A connection between lipid metabolism and the kidney has been formerly recognized [7]. For example, the kidney plays a crucial role in the catabolism of high-density lipoproteins (HDL) through filtration of delipidated apolipoprotein A1 (apoA-1) and small HDL by the glomerulus and reabsorption by the cubilin receptor [8]. Furthermore, CKD has been associated with the reduction in HDL cholesterol concentration, and the modification of the composition and function of this lipoprotein [9,10,11].

Although the onset of CKD is associated with dyslipidemia, a comprehensive assessment of circulating molecular lipid species associated with risk of CKD is scant. Recently, a study investigating plasma lipids associated with risk of CKD showed that higher baseline levels of multiple lipid classes, including sphingolipids, were associated with increased risk of CKD, independent of age, sex, body mass index, diabetes, and hypertension [12]. Sphingolipids are transported in the circulation incorporated into lipoproteins: low-, intermediate- and very low-density Apo B-containing lipoproteins (LDL, IDL, VLDL) and HDL, along with phospholipids, triglycerides, and cholesterol [13]. The abundant sphingolipids in the circulation are sphingomyelin, glycosphingolipids (glucosylceramide, lactosylceramide (Lact-Cer), and gangliosides) and ceramide. The plasma bioactive molecule sphingosine 1-phosphate (S1P) is mostly bound to HDL particles, particularly the small HDL3 particles, and about one third of plasma S1P is transported in the circulation bound to serum albumin [14].

Mechanisms by which sphingolipids in the liver are incorporated into lipoproteins, or how sphingolipids effluxed from tissues are transported to the liver, are mostly unknown. Microsomal triglyceride transfer protein (MTTP) and ATP-binding cassette A1 (ABCA1) have been shown as critical determinants of plasma ceramide, sphingomyelin, and hexosylceramide (Hex-Cer) levels [15,16]. However, the transport mechanisms of Lact-Cer, dihydroceramide, sphingoid bases and their phosphates from tissue cells into the liver or into macrophages, and their incorporation into lipoproteins, are still unidentified, but were shown to be independent of MTTP and ABCA1 [15,16]. Due to alterations in the lipid/sphingolipid metabolism, changes in the content of sphingolipids in the different lipoprotein particles may not reflect the measurement of sphingolipids in total plasma. A study comparing the lipidomic profile of LDL in patients with nondiabetic advanced renal disease and no evidence of cardiovascular disease (CVD) to that of age-matched controls showed that, in the renal disease patients, levels of triglycerides were increased, and levels of ceramides were decreased, while the total lipid and cholesterol content in LDL were unchanged [17].

There is mounting evidence supporting the role of sphingolipid metabolism defects in the pathogenesis and progression of renal disease [18]. We have recently reported differences in plasma levels of sphingolipids between normal healthy controls and diabetic patients with a normal albumin excretion rate (AER) or macroalbuminuria. Notably, we showed that, whereas the levels of LDL-cholesterol in the two diabetic groups were lower than in the control group, the levels of all measured ceramide and sphingomyelin species carried by LDL in the diabetic patients with macroalbuminuria (D-MA) were higher compared to controls [19]. We postulated that the residual cardiometabolic risk present in diabetes and not addressed by statin therapy may result from circulating sphingolipids.

In this study, both for plasma and isolated lipoproteins, we compared the levels of sphingolipids (ceramides, sphingomyelins, and sphingoid bases and their phosphates) and glycosphingolipids in healthy controls to those of patients with chronic kidney disease with diabetes (D-MA) and without diabetes (ND-CKD). We aimed to determine whether the differences observed were associated with the presence of renal damage or with diabetes, or whether both processes impacted circulating sphingolipid levels.

## 2. Methods

### 2.1. Research Design and Study Participants

Participants were recruited at the VA and MUSC Clinics of Endocrinology/Diabetes and Nephrology and from the staff at both institutions. Fasting blood samples were collected in EDTA for lipoprotein isolation and for plasma measurement of lipids, sphingolipids, and apolipoproteins A-I and B-100 from all participants. A total of 40 healthy controls with normal kidney function (eGFR > 60 mL/min), HbA1c < 6%, total cholesterol < 200 mg/dL, LDL-cholesterol < 130 mg/dL, triglycerides < 150 mg/dL, and HDL-Cholesterol > 40 mg/dL (men) and >50 mg/dL (women), and taking no medication used to treat any risk factor for diabetes and CVD disease were recruited from the staff of both institutions.

A total of 34 patients, previously diagnosed with type 2 diabetes and having macroalbuminuria (D-MA; AER > 300 mg/g creatinine), and 34 non-diabetic patients with chronic kidney disease (ND-CKD; eGFR < 59 mL/min but >15 mL/min, HbA1c < 5.6%, and no prior history of diabetes) were also recruited. In D-MA participants, 50% (17/34) had a recent eGFR <59 mL/min, while 53% (18/34) of the ND-CKD participants had a recent AER >300 mg/g creatinine. All participants had eGFR >15 mL/min.

The IRB at the Medical University of South Carolina (MUSC) approved the sample collection procedures. Written informed consent was obtained from all participants. Quantitative differences in circulating sphingolipids carried by plasma or isolated lipoproteins were compared between a group of healthy subjects and two groups of patients with established kidney disease, one of them with associated diabetes.

### 2.2. Lipoprotein Isolation

As previously described [19], lipoproteins (VLDL + IDL, LDL, HDL2, and HDL3) were isolated from the EDTA-plasma samples, and total protein and apolipoprotein A-I and B-100 concentrations [20] were determined in all plasma and isolated lipoprotein fractions.

### 2.3. Sphingolipid Extraction and Analysis

Mass spectroscopy was used to measure the concentration of individual species of five classes of sphingolipids: ceramides, sphingoid bases (sphingosine and dihydrosphingosine and their phosphates (S1P and dihydrosphingosine 1-phosphate), sphingomyelin, and the glycosphingolipids Lact-Cer and Hex-Cer in plasma and isolated lipoprotein, as previously described [19,21]. For ceramide, Hex-Cer, and Lact-Cer analysis, 400 µg protein from each individual sample was analyzed, and for sphingomyelin, 15 µg of VLDL, 25 µg of LDL, and 50 µg of each of HDL2 and HDL3 were analyzed. The adequate amount of protein needed for sphingomyelin (the most abundant sphingolipid) to be within the assay range was pre-determined in pilot studies. Results were reported as pmol/mL plasma.

### 2.4. Statistical Analysis

The study demographics and clinical characteristics were summarized for the entire cohort as well as stratified by group. To compare characteristics between study groups, the Kruskal–Wallis test was utilized for continuous variables and the Chi-Square test for categorical variables. Parameter effect sizes between groups are presented as Cohen’s *d*, calculated using the mean difference in log_10_ transformed data with pooled standard deviations. Sphingolipid levels are presented as geometric means and the associated standard errors. Cross-sectional comparisons between groups were conducted using analysis of variance models (ANOVA) and, when significant, pairwise between-group comparisons of interest were examined. A priori comparisons were of the healthy controls to the two disease groups (ND-CKD and D-MA) and between the two disease groups themselves. Significance levels of comparisons are noted in the study tables (*p* < 0.05, *p* < 0.01, *p* < 0.001). Species-specific effect sizes of C16:0, C18:0, C20:0, C22:0, C24:0, C24:1, and C26:0 in each class of sphingolipid were analyzed. All statistical analyses were performed using the SAS system version 9.4 (SAS Institute, Cary, NC, USA).

## 3. Results

### 3.1. Participant Characteristics

Participant characteristics across the three patient groups are presented in Table 1. Overall, participants were primarily males (62.3%; n = 66) and had an average age of 51.0 (SD = 11.9). Patients with D-MA were older than healthy controls and ND-CKD patients (pairwise *p* < 0.01). The gender and race distribution were similar across the three groups. The AER measurements compared to controls were markedly increased in patients with D-MA and patients with ND-CKD (pairwise *p* < 0.01). The levels of AER were elevated in patients with D-MA and ND-CKD compared to controls (pairwise *p* < 0.001). GFR was significantly lower in patients with ND-CKD and D-MA when compared to controls (pairwise *p* < 0.05). Hemoglobin A1c was significantly higher in the group with D-MA when compared to the controls and the ND-CKD group (pairwise *p* < 0.01). Total cholesterol and LDL-cholesterol levels were significantly elevated in the ND-CKD patients as compared to the other groups, and HDL-cholesterol levels in patients with ND-CKD were higher than in patients with D-MA (pairwise *p* < 0.01). Lastly, triglycerides were within normal limits in controls but higher than normal in patients with D-MA and patients with ND-CKD.

### 3.2. Ceramide and Sphingomyelin in Lipoproteins

**Figure 1:**
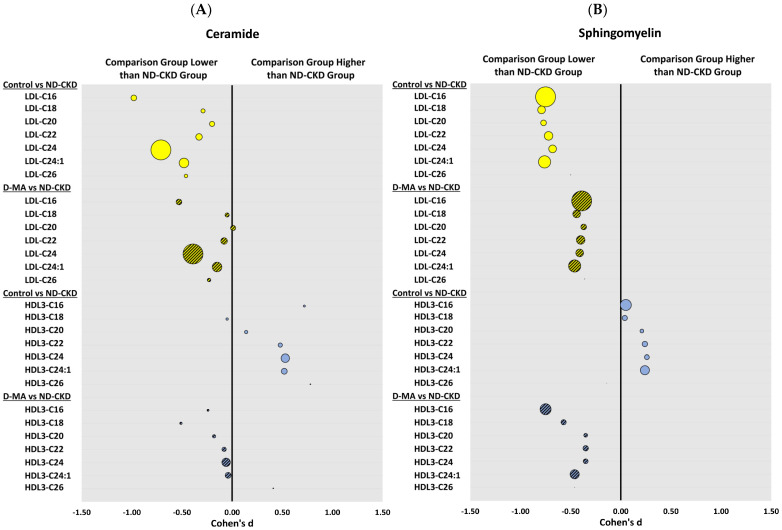
Between-group effect sizes for lipoprotein ceramide species (**A**) comparing the group of normal controls (Control) and the group of patients with type 2 diabetes and macroalbuminuria (D-MA) to non-diabetic patients with chronic kidney disease (ND-CKD). (**B**) Between-group effect sizes are presented for lipoprotein sphingomyelin species levels comparing the group of normal controls (Control) and the group of patients with type 2 diabetes and macroalbuminuria (D-MA) to non-diabetic patients with chronic kidney disease (ND-CKD). Mean difference in log_10_ transformed data and pooled standard deviations were used to calculate between-group effect sizes, and they are presented as Cohen’s d on the x-axis. The magnitude of the geometric mean level of the sphingolipid species carried by the corresponding lipoproteins (LDL and HDL3) in the ND-CKD group are reflected by the bubble size. Higher levels have larger bubble sizes. Yellow (LDL), blue (HDL), solid color (Control), stripes (D-MA).

Figure 1A,B depicts the effect size (Cohen’s d) of ceramide species (A) and sphingomyelin species (B) carried by LDL and HDL2 between healthy control participants (controls) and patients with ND-CKD, and between patients with D-MA and patients with ND-CKD. As shown in Figure 1A, LDL levels of C16:0 ceramide and C24:0 ceramide, the most abundant ceramide species in LDL (three to seven times higher than any of the other ceramide species), were significantly lower in the control group than in the ND-CKD group. The levels of these two ceramide species were also lower in patients with D-MA compared to the ND-CKD group, but the difference did not reach statistical significance. Interestingly, although the trend observed for ceramide species carried by VLDL/IDL was similar, no statistical differences among the groups were detected for any of the ceramide species, and the levels in the D-MA and ND-CKD groups were quite similar, while higher than those obtained in the controls. (Table 2).

In contrast to LDL, the levels of ceramide species carried by HDL3 and HDL2 were higher in controls than in patients with ND-CKD. There is no significant difference between ceramide levels carried by HDL2 and HDL3 in D-MA and those with ND-CKD patients (Figure 1A and Table 2).

Sphingomyelin levels carried by LDL and VLDL were significantly lower in controls than in patients with ND-CKD, as shown in Figure 1B for LDL and in Table 3 for both LDL and IDL/VLDL. Interestingly, the levels of sphingomyelin carried by LDL and IDL/VLDL in patients with D-MA were numerically higher than those carried by the same lipoproteins obtained from normal controls, but due to the small number of patients, statistically significant differences, either when compared with the ND-CKD group or with controls, were not detected. The levels for D-MA were between the levels found in ND-CKD and controls.

In contrast, the levels of sphingomyelin carried by HDL2 and HDL3 were significantly lower across all species in D-MA patients when compared to normal controls or ND-CKD patients, but no significant differences were present in sphingomyelin species carried by HDL2 and HDL3 isolated from controls when compared to those isolated from ND-CKD patients. In general, the sphingomyelin levels carried by HDL2 and HDL3 were higher in controls than in ND-CKD patients, but the differences were small and did not reach statistical significance. Patients with D-MA had significantly lower levels of C16:0 sphingomyelin in both HDL2 and HDL3 as well as significantly lower levels of C24:1 sphingomyelin in HDL2 when compared with patients with ND-CKD (Figure 1B and Table 3).

### 3.3. Glycosphingolipids in Lipoproteins

**Figure 2:**
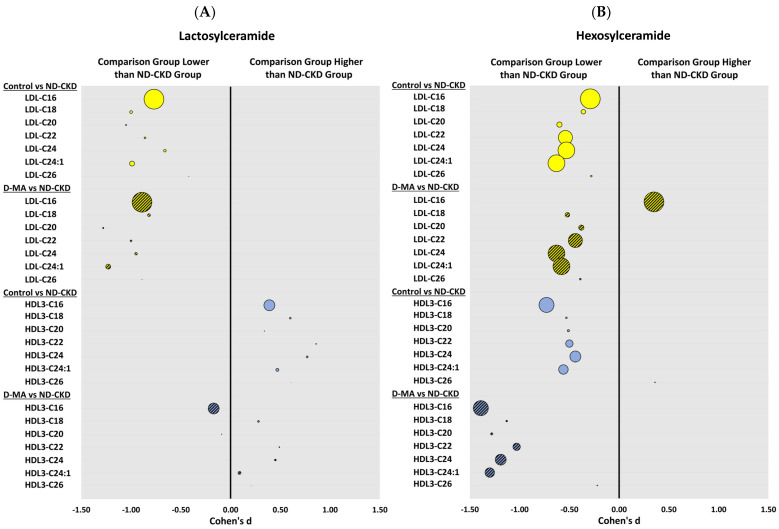
Between-group differences are presented for (**A**) lactosylceramide and (**B**) hexosylceramide species levels comparing the group of normal controls (Control) and the group of patients with type 2 diabetes and macroalbuminuria (D-MA) to non-diabetic patients with chronic kidney disease (ND-CKD). Mean difference in log10 transformed data and pooled standard deviations were used to calculate between-group effect sizes, and they are presented as Cohen’s d on the x-axis. The magnitude of the geometric mean level of the sphingolipid species carried by the corresponding lipoproteins (LDL and HDL3) in the ND-CKD group is reflected by the bubble size. Higher levels have larger bubble sizes. Yellow (LDL), blue (HDL), solid color (Control), stripes (D-MA).

The levels of Lact-Cer species carried by Apo B-containing lipoproteins (LDL and VLDL/IDL) isolated from both control participants and D-MA patients were significantly lower than in ND-CKD patients. The levels of Lact-Cer species carried by Apo B-containing lipoproteins in controls and in patients with D-MA were not significantly different. (Figure 2A and Table 4).

The levels of Lact-Cer carried by HDL3 were significantly higher in controls (C18:0, C22:0, C24:0 and C26:0 Lact-Cer) than in patients with ND-CKD. HDL3 C22:0 Lact-Cer was the only species significantly higher in patients with D-MA than in patients with ND-CKD. Levels of Lact-Cer carried by HDL2 were similar in the 3 groups except for C16:0 Lact-Cer, which was significantly lower in patients with D-MA patients than in controls (Table 4).

The results of the Hex-Cer levels are presented in Figure 2B. The levels of Hex-Cer species carried by LDL, except for C16:0 Hex-Cer, were significantly lower in controls and in patients with D-MA than in patients with ND-CKD. C16:0 Hex-Cer carried by LDL was significantly higher in patients with D-MA than in normal controls. The levels carried by IDL/VLDL were similar in the three groups (Table 5).

The most important differences in Hex-Cer levels among the three groups were observed in HDL2 and HDL3. The levels of all Hex-Cer species carried by HDL3 and HDL2 were significantly lower in both the controls and patients with D-MA, when compared to ND-CKD patients. Interestingly, however, the levels of the C16:0 and C24:0 Hex-Cer, the two most abundant species of Hex-Cer within HDL2 and HDL3, were significantly lower in patients with D-MA than in controls.

### 3.4. Sphingoid Bases and Their Phosphates in Lipoproteins

**Figure 3:**
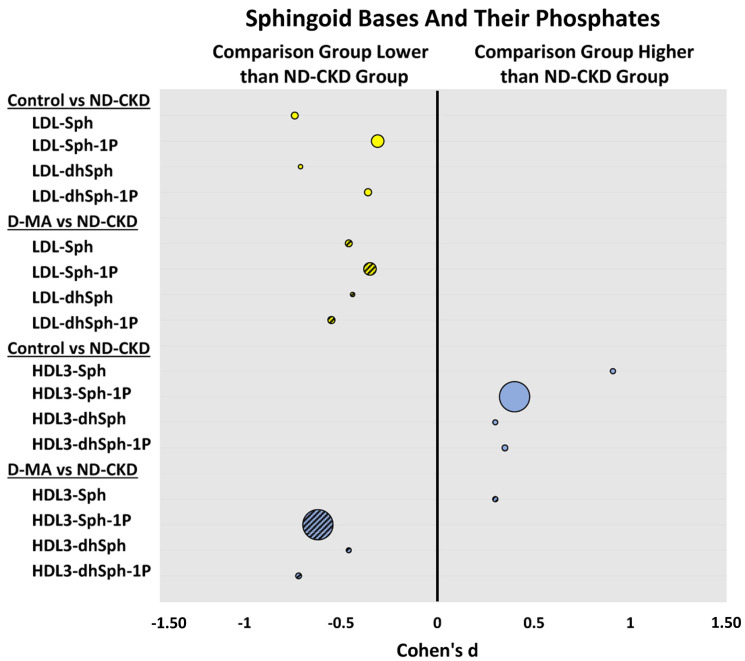
Between-group effect sizes are presented for the levels of sphingoid bases and their phosphates comparing the group of normal controls (Control) and the group of patients with type 2 diabetes and macroalbuminuria (D-MA) to non-diabetic patients with chronic kidney disease (ND-CKD). Mean difference in log10 transformed data and pooled standard deviations were used to calculate between-group effect sizes, and they are presented as Cohen’s d on the x-axis. The magnitude of the geometric mean level of the sphingolipid species carried by the corresponding lipoproteins (LDL and HDL3) in the ND-CKD group are reflected by the bubble size. Higher levels have larger bubble sizes. Sphingoid bases: sphingosine (Sph) and dihydrosphingosine (dhSph), and their phosphates: sphingosine 1-phosphate (Sph-1P), and dihydrosphingosine 1-phosphate (dhSph-1P). Yellow (LDL), blue (HDL), solid color (Control), stripes (D-MA).

In LDL and VLDL/IDL, the levels of sphingosine and dihydrosphingosine were lower in both the controls and patients with D-MA than in patients with ND-CKD. In contrast, significantly lower levels of sphingoid bases and their phosphates were carried by HDL2 and HDL3 in patients with D-MA when compared to normal controls. The levels of sphingosine within HDL3 and HDL2 were significantly higher in the controls compared to the ND-CKD group. S1P within HDL3 was significantly lower in D-MA than in ND-CKD patients. (Figure 3 and Table 6). 

### 3.5. Plasma Levels of Sphingolipids

Plasma levels of ceramides, sphingomyelin, Lact-Cer, and Hex-Cer are presented in Table 7. Plasma levels of C18:0 ceramide were significantly higher in both groups of renal disease, D-MA and ND-CKD, than those in normal controls. Levels of C20:0 ceramide in patients with ND-CKD were also significantly increased when compared with the levels in normal controls. As for sphingomyelin, the only significant difference observed among the three groups was in the plasma levels of C16:0 sphingomyelin, which were significantly increased in ND-CKD compared to controls. The levels of plasma glycosphingolipids were also altered in the ND-CKD group. All species of plasma Hex-Cer were markedly increased in the ND-CKD group when compared to those in the plasma of controls and in the plasma of the D-MA group. In contrast, the levels of very long-chain Lact-Cer species (C22:0; C24:0, and C26:0) were significantly decreased in the ND-CKD and D-MA groups when compared to those in controls. Lastly, levels of all sphingoid bases and their phosphates were markedly reduced in both ND-CKD and D-MA (Table 7).

## 4. Discussion

This is the first time that the levels of sphingolipids carried by circulating lipoproteins in patients with D-MA and in ND-CKD are compared. Our results clearly show both similarities and remarkable differences in the circulating sphingolipids between these two groups of patients with kidney disease.

The levels of ceramide and sphingomyelin species carried by LDL in ND-CKD patients were significantly higher than in controls (Figure 1 and Table 1 and Table 2). However, the levels of ceramide and sphingomyelin carried by LDL in the D-MA group, while also higher than in controls, were not statistically different from those measured in controls or ND-CKD patients, with levels in the middle of those in the two groups. This is an interesting finding since we recently documented that, when comparing the levels of sphingomyelin and ceramide carried by LDL in two groups of patients with diabetes, one group with normoalbuminuria and the other with macroalbuminuria [19], both sphingomyelin and ceramide levels were higher in the group with macroalbuminuria compared to the group with normoalbuminuria. When compared to normal controls, the LDL from patients with diabetes with normoalbuminuria carried more ceramide but less sphingomyelin than controls [19,22]. The higher level of ceramide carried by LDL in diabetes is surprising since LDL-C levels in those patients, even in those with macroalbuminuria, were lower than in control subjects. These data suggest that statin therapy lowers LDL-C but not ceramide levels, since the patients with diabetes were more likely to be on statin therapy (75%) than the control group (25%). These data also support previous reports showing that statin therapy does not lower ceramide levels in LDL, just cholesterol levels [19,23], and suggest a possible effect of diabetes per se on sphingomyelin levels, since the levels of sphingomyelin carried by LDL, unlike ceramide levels, were lower than in the controls. A possible explanation is that diabetes may activate neutral sphingomyelinase, likely at the liver level, leading to an increase in ceramide and to a decrease in sphingomyelin. Activation of sphingomyelinase by generating ceramide and creating a cascade of bioactive lipids are known to contribute to CVD risk [24], which strongly suggests that one of the important residual risk factors for the development of CVD and other complications of diabetes, not addressed by statin therapy, is likely to be the increased levels of sphingolipids carried by Apo B-containing lipoproteins.

The levels of triglycerides were higher in both groups of kidney disease, with and without diabetes, than in the control group, but the difference failed to reach statistical significance. All classes of sphingolipids carried by VLDL were slightly or significantly higher in both groups of kidney disease than in controls, except for C16:0 and C18:0 Hex-Cer, which were lower but without reaching statistical difference in the ND-CKD group. In general, however, the majority of sphingolipid species carried by VLDL were not significantly increased compared to controls, except for sphingomyelin and Lact-Cer in the ND-CKD group. Significant differences in sphingolipids carried by VLDL between the two groups of kidney disease were not observed. Interestingly, both ceramide and sphingomyelin levels in VLDL were higher in patients with D-MA than in normal controls regardless of lower levels of cholesterol in the D-MA group.

The levels of glycosphingolipids (Lact-Cer and Hex-Cer) carried by LDL were also significantly increased in the ND-CKD group when compared to the normal controls, but, in contrast, the LDL glycosphingolipid levels in the D-MA group were quite similar to those observed in normal controls, except for C16:0 Hex-Cer, which was significantly higher in patients with D-MA than in normal controls. Interestingly, and in contrast to the levels of ceramide carried by VLDL/IDL, which were quite like those of normal controls, the levels of glycosphingolipids carried by VLDL/IDL were higher in both groups with kidney disease compared to normal controls, although statistical significance was only attained in the ND-CKD group. The levels in the D-MA group were between the levels in the control group and the ND-CKD group. The levels followed a trend similar to those observed for triglyceride levels.

The levels of sphingolipids carried by HDL, both HDL3 and HDL2, are quite striking and they confirm not only our previous findings showing that diabetes leads to reduced efflux of ceramide, sphingomyelin, and Lact-Cer into HDL [19], but also that, when renal damage occurs, the levels of these sphingolipids carried by HDL increase, but remain lower than those carried by HDL in normal controls. As we previously postulated for diabetes, the marked decrease of ceramide, Lact-Cer, and sphingomyelin carried by HDL may derive from diabetes-induced changes in the activity of the enzymes involved in the intracellular regulation of sphingolipids, resulting in decreased production, decreased efflux, or both. Most of these changes are likely induced by reactive oxygen species and inflammatory mediators. Kidney damage may further accentuate the dysregulation of sphingolipid metabolism, but by differentially upregulating production, promote the slight increase in levels observed in the two groups of kidney disease. Increased accumulation of sphingolipids in the diabetic kidney has been well documented [25] and it is a major cause of lipotoxicity [26,27,28].

It is noteworthy to mention that the presence of diabetes per se did not decrease the levels of Hex-Cer carried by HDL. We have previously shown that the levels of HDL Hex-Cer in patients with diabetes but without macroalbuminuria were slightly increased when compared to normal controls, although the differences did not reach statistical significance [19]. Notably, when macroalbuminuria develops in patients with type 2 diabetes, Hex-Cer levels carried by both HDL2 and HDL3 were decreased, and the decrease was significant for the most abundant species of Hex-Cer carried by HDL2. In the absence of diabetes, however, renal damage strongly impacted the levels of Hex-Cer carried by HDL, leading to a significant increase in the Hex-Cer levels carried by HDL (HDL2 and HDL3). This is quite a remarkable finding allowing the levels of Hex-Cer carried by HDL to completely differentiate D-MA from other types of chronic renal disease.

Although abundant information is available concerning the synthesis, metabolism, and signaling mechanisms of sphingolipids [29,30,31,32,33], little is known about their transport in the circulation. ABCA1 deficiency was found to be associated in humans and mice with low Hex-Cer levels [16]. It is also well known that ABCA1 participates in the transport of cholesterol and phospholipids to HDL and is critical in generating HDL, which is a potent acceptor for the efflux of Hex-Cer [16]. A more recent study, however, suggests that ABCA1 is not directly involved in the efflux of Hex-Cer [34]. ABCC10, which is expressed in several tissues including the kidney [35], was identified as participating in Hex-Cer biosynthesis and affecting its efflux. Interestingly, it was also shown that although ABCC10 influences Hex-Cer synthesis, it has no direct effect on glucosylceramide synthase (GCS). It may impact Hex-Cer synthesis by affecting substrate availability or promoting substrate removal from the GCS enzyme. Budani et al. have recently shown that several ABC transporters potentially acting as glucosylceramide flippases may differentially regulate Hex-Cer synthesis [36]. Therefore, we postulate that renal damage and diabetes may differentially affect some of the glucosylceramide flippases, leading to opposite effects on Hex-Cer efflux. This needs to be verified and will certainly open an interesting field of research with possible repercussions on approaches to treatment.

Lastly, some studies in type 2 diabetes, have reported increased plasma levels of S1P [37,38]. Our present data as well as data we have previously reported [19] show that the levels of S1P and other sphingoid bases (both in plasma and in HDL fractions) are decreased in type 2 diabetic patients. Plasma S1P concentrations include concentrations in the lipoproteins plus those bound to albumin; all other sphingolipids are carried only by lipoproteins [14]. Other studies in type 2 diabetes have found decreased levels of S1P carried by HDL, which were also associated with impaired HDL function [39,40].

The increase in sphingoid bases and their phosphates carried by Apo B-containing lipoproteins observed in patients once they developed kidney disease may result from overproduction of these sphingolipids induced by kidney damage. These findings were supported by our present data that clearly show that in ND-CKD patients the levels of the sphingoid bases and their phosphates carried by LDL and VLDL are higher than those observed in D-MA. In contrast, the efflux of the sphingoid bases and their phosphates into HDL2 and HDL3, while reduced by the presence of diabetes, as previously shown [21], is also observed in patients with kidney damage without diabetes, suggesting that the decrease in efflux observed in D-MA and ND-CKD are likely mediated by pro-inflammatory mediators released both in kidney damage and diabetes since both diseases have a strong inflammatory component. Studies examining the role of sphingolipids/glycosphingolipids in oxidative stress and the possible use of antioxidants to counteract some of the oxidative stress damage in diabetic complications as well as in chronic diseases with an inflammatory component are still needed [41].

In summary, our data show that the levels of ceramide and sphingomyelin carried by Apo B-containing lipoproteins are higher in patients with ND-CKD and D-MA than in controls. In contrast, both ceramide and sphingomyelin carried by HDL are decreased in patients with ND-CKD and D-MA, but the decrease, mainly of sphingomyelin, is more accentuated in D-MA. This finding suggests that diabetes greatly impairs sphingomyelin efflux from tissues and/or sphingomyelin incorporation into HDL. Glycosphingolipids (both Lact-Cer and Hex-Cer) carried by Apo B-containing lipoproteins are increased in ND-CKD; however, the levels in D-MA are similar to those of controls, except for C16-Hex-Cer. The levels of Hex-Cer carried by HDL distinctly differentiate patients with D-MA from those with ND-CKD. Whereas Hex-Cer levels in ND-CKD patients are significantly higher than in controls, in D-MA patients, Hex-Cer levels are significantly lower than in controls and in ND-CKD patients.

The data in this paper confirm that the information provided by total plasma levels of sphingolipids misses very important changes that occur at the lipoprotein level. In patients with ND-CKD, significantly increased levels of ceramides carried by LDL, including C24:0 ceramide, the most abundant ceramide species carried in the circulation, are totally missed when only plasma levels are measured. The same occurs for the same ceramide species carried by HDL3 and HDL2 since the levels are significantly low. The decrease in ceramide species carried by HDL2 and HDL3 will completely preclude detecting the marked increase of the same ceramide species carried by LDL. The same occurs for sphingomyelin where the marked and significant increase in sphingomyelin carried by the Apo B-containing lipoproteins in patients with ND-CKD is totally missed when plasma levels are the only available measurement. Only when the increase or reduction in the sphingolipid species carried by Apo B-containing lipoproteins and HDL coincides does the plasma measurement have value, and that, unfortunately, occurs very rarely. Hex-Cer levels carried by HDL clearly differentiates ND-CKD from D-MA patients, but that would have been totally missed if only plasma levels were obtained. To really understand the relevance of circulating sphingolipids in the pathophysiology of disease states, circulating sphingolipids need to be measured in lipoproteins and plasma, not just in plasma.

## 5. Study Limitations

The small number of patients studied ([34,35,36,37,38,39,40] per group), and the fact that patients with chronic kidney disease were older than the controls, constitute the major limitation of this study. The magnitude of the changes observed may also be limited by the fact that the patients had good glucose and lipid control. A small number of patients per group precluded covariate analyses and, therefore, the study of the impact of age, race, body mass index (BMI), or statin therapy on the data. Validation in a large prospective and/or cross-sectional study is definitively required.

## 6. Conclusions

Together with other published studies examining the distribution of sphingolipids carried by lipoproteins, this study will open a new field of research and fill an obvious gap in our present knowledge. In the vasculature and in the majority of organs, circulating lipoproteins play a crucial role in the uptake/removal of lipids, but their function differs significantly among the different tissues, leading to marked differences in metabolism and physiopathology. Considering the important role sphingolipids play in major cell biological responses and cell signaling pathways, we can easily postulate that changes in the distribution of sphingolipids and glycosphingolipids carried by lipoproteins will modulate the development and progression of many diseases and change the therapeutic approaches for chronic inflammatory diseases. Our work represents one of the first steps to address a considerable gap in this important research and clinical area.

## Figures and Tables

**Table 1:** Demographic and clinical characteristics between study groups.

Characteristic	Study Group			*p*-Value
	Non-Diabetic CKD (ND-CKD)	Diabetes with MA * (D-MA)	Controls *	
	(n = 34)	(n = 34)	(n = 40)	
Gender Male % (n)	61.8% (21)	70.6% (24)	55.0% (22)	0.41
Race White % (n)	56.9% (19)	56.9% (19)	42.5% (17)	0.41
Age (years)	47.6 (15.3)	56.1 (7.5)	49.4 (10.4)	0.03
LDL Cholesterol mg/dl	133.6 (61.1)	104.5 (45.7)	118.2 (34.2)	0.02
HDL Cholesterol mg/dl	54.6 (22.6)	41.0 (11.6)	50.0 (16.6)	<0.01
Triglycerides mg/dl *	145 (104,224)	163 (88,232)	101 (68,178)	0.05
Total Cholesterol mg/dl	235.3 (76.1)	184.0 (56.8)	192.3 (41.2)	<0.01
HbA1c %	<6.0	8.4 (8.2)	<6.0	<0.01
HbA1c (mmol/mol)	37.7 (3.6)	68.5 (20.9)	34.4 (4.8)	<0.01
AER (mg/24 H) **	1208 (103,1921)	605 (475,1079)	4.9 (2.7,8.7)	<0.01
GFR mL/min/1.73 m^2^	48.7 (22.0)	61.9 (26.2)	88.5 (17.8)	<0.01

* Data reported in this table for these two groups were previously described in Reference [19] and are now being compared to data obtained in patients with CKD but without diabetes. Continuous data shown as group level means and associated standard deviation. ** AER and Triglycerides shown as median (inner quartile range). Categorical variables shown as % and (n). *p* values are calculated using Kruskal–Wallis test statistic for continuous variables and Chi-Square test statistic for categorical variables.

**Table 2:** Lipoprotein ceramide species levels (pmol/mL plasma).

Ceramide	Group			Cohen’s d		
	Control	D-MA	ND-CKD	Control vs. ND-CKD	Control vs. D-MA	D-MA vs. ND-CKD
**LDL C16:0**	31.3 (5.1)	46.8 (7.4)	74.8 (11.2) # **	−0.98	−0.44	−0.53
**LDL C18:0**	28.7 (6.9)	38.9 (6.2)	41.0 (7.8)	−0.29	−0.26	−0.05
**LDL C20:0**	43.8 (13.7)	61.7 (12.6)	60.7 (15.7)	−0.20	−0.23	0.01
**LDL C22:0**	72.6 (10.7)	90.2 (15.0)	97.2 (15.7)	−0.33	−0.24	−0.08
**LDL C24:0**	467 (76.1)	598.2 (109.1)	864.1 (122.5) *	−0.71	−0.25	−0.39
**LDL C24:1**	128.7 (21.7)	177.6 (33.8)	207.7 (37.4)	−0.48	−0.31	−0.15
**LDL C26:0**	17.7 (3.4)	22.4 (4.6)	29.9 (5.1)	−0.46	−0.20	−0.23
**HDL3 C16:0**	20.6 (3.9)	9.8 (1.2) **	11.4 (1.2) *	0.72	0.86	−0.24
**HDL3 C18:0**	14.7 (3.3)	9.3 (1.6)	15.0 (2.4) #	−0.05	0.38	−0.51
**HDL3 C20:0**	33.1 (9.4)	21.3 (4.2)	26.2 (5.3)	0.14	0.30	−0.18
**HDL3 C22:0**	59.9 (9.3)	41.0 (4.8) ^	43.1 (4.4)	0.48	0.53	−0.08
**HDL3 C24:0**	242.6 (41.3)	150.8 (23.9) ^	159.1 (21.1) ^	0.53	0.55	−0.06
**HDL3 C24:1**	113.7 (17.6)	76.2 (9.9) ^	78.5 (9.2) ^	0.52	0.54	−0.04
**HDL3 C26:0**	4.6 (0.8)	3.4 (0.5) ƚ	2.4 (0.4) *	0.78	0.37	0.41
**VLDL C16:0**	7.6 (1.7)	11.9 (1.4)	11.3 (2.2)	−0.34	−0.46	0.06
**VLDL C18:0**	9.1 (2.7)	14.6 (2.5)	10.8 (2.6)	−0.11	−0.35	0.25
**VLDL C20:0**	26 (8.6)	43.8 (9.3)	31.5 (8.9)	−0.11	−0.33	0.23
**VLDL C22:0**	19 (3.1)	21 (3.1)	19.2 (3.3)	−0.02	−0.11	0.09
**VLDL C24:0**	75.4 (13.3)	106 (19.5)	116.1 (21.1)	−0.42	−0.33	−0.09
**VLDL C24:1**	39.3 (6.6)	50.3 (8.3)	46.2 (8.5)	−0.16	−0.26	0.09
**VLDL C26:0**	2 (0.4)	2.6 (0.6)	2.6 (0.5)	−0.21	−0.2	0.00
**HDL2 C16:0**	18.8 (3.5)	10.5 (1.4) *	12.4 (1.8)	0.44	0.65	−0.21
**HDL2 C18:0**	14.4 (3.1)	9.8 (1.8)	12.8 (1.9)	0.11	0.34	−0.27
**HDL2 C20:0**	23.8 (6.5)	16 (3.6)	18.8 (4.1)	0.17	0.28	−0.13
**HDL2 C22:0**	43.1 (6.9)	31.4 (4.9)	30.7 (4.5)	0.39	0.36	0.03
**HDL2 C24:0**	322.8 (56.1)	205.1 (36.1)	195.5 (33.8) ^	0.51	0.47	0.05
**HDL2 C24:1**	77 (13.5)	52 (9.5)	51.6 (8.8)	0.41	0.39	0.01
**HDL2 C26:0**	8.5 (1.6)	6.1 (1.1)	3.7 (0.7) *	0.75	0.32	0.45

^ *p* < 0.05, * *p* < 0.01, ** *p* < 0.001 compared to Control. # *p* < 0.05, ƚ *p* < 0.01, ǂ *p* < 0.001 compared to Diabetes. Data are depicted as geometric means and associated standard errors. Significance is determined utilizing linear regression models. Overall regression f-tests were used to determine species significance and pairwise contrasts used to compare diabetes groups to controls. Between-group effect sizes are presented as Cohen’s d, calculated using mean difference in log_10_ transformed data and pooled standard deviations.

**Table 3:** Lipoprotein sphingomyelin species levels (pmol/mL plasma).

Sphingomyelin	Group			Cohen’s d		
	Control	D-MA	ND-CKD	Control vs. ND-CKD	Control vs. D-MA	D-MA vs. ND-CKD
**LDL C16:0**	22,414 (4258)	33,922 (4901)	47,479 (8242) *	−0.75	−0.43	−0.39
**LDL C18:0**	3479 (644)	5094 (777)	7356 (1111) *	−0.79	−0.39	−0.44
**LDL C20:0**	2008 (354)	2980 (455)	4077 (635) *	−0.77	−0.41	−0.37
**LDL C22:0**	4651 (820)	6415 (1010)	9094 (1434) *	−0.72	−0.33	−0.4
**LDL C24:0**	3919 (706)	5299 (752)	7404 (1172) ^	−0.68	−0.32	−0.41
**LDL C24:1**	8470 (1683)	12,299 (1846)	18,089 (2816) *	−0.76	−0.37	−0.46
**LDL C26:0**	12.3 (3.5)	17.1 (3.2)	25.8 (6.2)	−0.50	−0.24	−0.36
**HDL3 C16:0**	15,055 (2306)	9336 (1141) *	14,775 (1385) ƚ	0.05	0.64	−0.75
**HDL3 C18:0**	3435 (484)	2479 (266) ^	3404 (314) #	0.04	0.49	−0.57
**HDL3 C20:0**	2151 (308)	1569 (153) ^	1889 (178)	0.21	0.48	−0.35
**HDL3 C22:0**	4353 (628)	3077 (285) ^	3711 (363)	0.24	0.53	−0.35
**HDL3 C24:0**	3594 (551)	2447 (239) ^	2981 (321)	0.26	0.55	−0.35
**HDL3 C24:1**	11,923 (1701)	7869 (841) *	10,263 (1012)	0.24	0.61	−0.46
**HDL3 C26:0**	10.2 (2.2)	8.1 (1.0)	11.8 (1.9)	−0.14	0.22	−0.46
**VLDL C16:0**	4376.4 (897)	6237 (714)	7559 (1319) ^	−0.51	−0.37	−0.24
**VLDL C18:0**	643.8 (128)	1013 (129.5)	1140 (201) ^	−0.54	−0.47	−0.14
**VLDL C20:0**	362.6 (70.3)	537.1 (70)	614.5 (111)	−0.50	−0.41	−0.16
**VLDL C22:0**	704 (134.3)	1011 (133.5)	1192 (209) ^	−0.51	−0.38	−0.19
**VLDL C24:0**	551 (105.4)	742.1 (84.9)	856.3 (149)	−0.43	−0.33	−0.18
**VLDL C24:1**	1650 (323.8)	2299 (242.5)	2797 (465) ^	−0.51	−0.37	−0.26
**VLDL C26:0**	0.6 (0.1)	0.8 (0.2)	1.2 (0.3) ^	−0.56	−0.31	−0.30
**HDL2 C16:0**	15,442 (2425)	8305 (1262) *	12,962 (1563) #	0.22	0.72	−0.58
**HDL2 C18:0**	2453.4 (372)	1405 (225) *	1985 (210)	0.29	0.64	−0.45
**HDL2 C20:0**	1242 (193.6)	743.9 (108.5) *	958.3 (107.6)	0.34	0.61	−0.35
**HDL2 C22:0**	2513 (385.8)	1465 (199.9) *	1944 (232)	0.34	0.66	−0.39
**HDL2 C24:0**	2317 (363.8)	1255 (176.7) *	1728 (232)	0.36	0.73	−0.41
**HDL2 C24:1**	5678 (858.5)	2928 (460) *	4464 (549) #	0.31	0.76	−0.53
**HDL2 C26:0**	11.4 (2.2)	5.2 (1.2) *	8.8 (1.5)	0.26	0.65	−0.45

^ *p* < 0.05, * *p* < 0.01, ** *p* < 0.001 compared to Control. # *p* < 0.05, ƚ *p* < 0.01, ǂ *p* < 0.001 compared to Diabetes. Data are depicted as geometric means and associated standard errors. Significance is determined utilizing linear regression models. Overall regression f-tests were used to determine species significance and pairwise contrasts used to compare diabetes groups to controls. Between-group effect sizes are presented as Cohen’s d, calculated using mean difference in log_10_ transformed data and pooled standard deviations.

**Table 4:** Lipoprotein lactosylceramide species levels (pmol/mL plasma).

Lactosylceramide	Group			Cohen’s d		
	Control	D-MA	ND-CKD	Control vs. ND-CKD	Control vs. D-MA	D-MA vs. ND-CKD
**LDL C16:0**	841.1 (121.2)	711.8 (118.9)	1514 (185) ǂ *	−0.77	0.19	−0.89
**LDL C18:0**	14.0 (2.4)	16.0 (2.8)	34.1 (4.8) ƚ **	−1.00	−0.14	−0.82
**LDL C20:0**	2.9 (0.5)	2.6 (0.9)	8.5 (1.6) ǂ **	−1.05	0.12	−1.28
**LDL C22:0**	7.2 (1.0)	6.8 (0.8)	15.8 (2.8) ǂ **	−0.86	0.08	−1.00
**LDL C24:0**	15.3 (2.4)	12.1 (1.6)	29.6 (5.6) ǂ *	−0.66	0.29	−0.95
**LDL C24:1**	42.3 (7.0)	36.0 (5.2)	99.7 (13.9) ǂ **	−0.99	0.18	−1.23
**LDL C26:0**	0.6 (0.1)	0.4 (0.1) ^	0.8 (0.2) ƚ	−0.42	0.53	−0.89
**HDL3 C16:0**	708.3 (133.1)	385.4 (73.6) ^	471.7 (100.5)	0.39	0.61	−0.17
**HDL3 C18:0**	25.6 (4.8)	18.7 (2.2)	15.5 (2.0) ^	0.60	0.39	0.28
**HDL3 C20:0**	2.4 (0.5)	1.6 (0.2)	1.7 (0.3)	0.34	0.46	−0.09
**HDL3 C22:0**	9.8 (1.8)	6.4 (0.7) ^	4.3 (0.7) # **	0.86	0.56	0.49
**HDL3 C24:0**	66.4 (14.6)	21.4 (2.3)	14.9 (2.4) *	0.77	0.48	0.45
**HDL3 C24:1**	66.4 (14.6)	43.5 (7.0)	39.3 (8.3)	0.47	0.45	0.09
**HDL3 C26:0**	0.4 (0.1)	0.2 (0.1) ^	0.1 (0.0) ^	0.61	0.51	0.21
**VLDL C16:0**	181.6 (40.2)	230.7 (52.6)	297.1 (66.1)	−0.39	−0.18	−0.19
**VLDL C18:0**	2.4 (0.5)	3.4 (0.7)	5.4 (1.0) *	−0.72	−0.29	−0.42
**VLDL C20:0**	0.2 (0.1)	0.4 (0.1)	0.6 (0.1) *	−0.72	−0.35	−0.41
**VLDL C22:0**	0.7 (0.1)	1 (0.1)	1.3 (0.2) ^	−0.55	−0.3	−0.32
**VLDL C24:0**	1.6 (0.3)	2.2 (0.3)	2.9 (0.6) ^	−0.50	−0.33	−0.25
**VLDL C24:1**	4.6 (1.1)	5.7 (1.2)	9.2 (1.8) ^	−0.57	−0.17	−0.41
**VLDL C26:0**	0 (0)	0 (0)	0.1 (0.0)	−0.49	−0.26	−0.29
**HDL2 C16:0**	823.6 (167)	444.6 (92.2) ^	831.8 (241.6)	−0.01	0.54	−0.42
**HDL2 C18:0**	16.6 (3.7)	11 (2.2)	15.3 (4.7)	0.06	0.35	−0.21
**HDL2 C20:0**	2.1 (0.5)	1.7 (0.2)	3.0 (0.7) #	−0.25	0.23	−0.49
**HDL2 C22:0**	6.5 (1.1)	4.8 (0.5)	5.9 (1.3)	0.08	0.39	−0.20
**HDL2 C24:0**	19.7 (3.4)	13.5 (1.6)	14.8 (3.0)	0.26	0.46	−0.10
**HDL2 C24:1**	46.5 (10.1)	30 (5.5)	44.9 (12.2)	0.02	0.39	−0.30
**HDL2 C26:0**	0.4 (0.1)	0.3 (0.1)	0.2 (0.1) ^	0.56	0.37	0.28

^ *p* < 0.05, * *p* < 0.01; ** *p* < 0.001 compared to Control Group. # *p* < 0.05, ƚ *p* < 0.01, ǂ *p* < 0.001 Compared to Diabetes Macroalbuminuria Group. Data are depicted as Geometric means and associated standard errors. Significance is determined utilizing linear regression models. Overall regression f-test were used to determine species significance and pairwise contrasts used to compare diabetes groups to controls. Between-group effect sizes are presented as Cohen’s d; calculated using mean difference in log_10_ transformed data and pooled standard deviations.

**Table 5:** Lipoprotein hexosylceramide species levels (pmol/mL plasma).

Hexosylceramide	Group			Cohen’s d		
	Control	D-MA	ND-CKD	Control vs. ND-CKD	Control vs. D-MA	D-MA vs. ND-CKD
**LDL C16:0**	461.2 (86.7)	838.8 (123.0) ^	614.7 (96.6)	−0.29	−0.62	0.35
**LDL C18:0**	22.9 (4.8)	18.1 (4.1)	34.9 (7.2) #	−0.36	0.19	−0.52
**LDL C20:0**	23.1 (4.6)	30.8 (4.8)	45.7 (9.1) ^	−0.60	−0.28	−0.38
**LDL C22:0**	179.5 (32.4)	209.8 (28.3)	312.4 (55.4) ^	−0.54	−0.17	−0.44
**LDL C24:0**	247.7 (49.1)	238.6 (35.9)	441.2 (81.8) #^	−0.53	0.04	−0.63
**LDL C24:1**	239.1 (43.3)	267.7 (37.0)	445.6 (74.2) #^	−0.63	−0.12	−0.58
**LDL C26:0**	4.3 (0.8)	4.1 (0.6)	5.7 (0.9)	−0.28	0.06	−0.39
**HDL3 C16:0**	117.5 (25.2)	81.1 (13.7)	256.4 (30.4) ǂ*	−0.73	0.40	−1.39
**HDL3 C18:0**	2.9 (0.6)	1.7 (0.3) ^	5.4 (1.0) ǂ^	−0.53	0.54	−1.13
**HDL3 C20:0**	5.6 (1.2)	4.0 (0.5)	9.6 (1.0) ǂ^	−0.51	0.42	−1.28
**HDL3 C22:0**	54.0 (11.1)	44.7 (6.0)	89.2 (8.9) ǂ^	−0.50	0.25	−1.03
**HDL3 C24:0**	123.0 (24.0)	85.0 (10.8)	189.3 (20.3) ǂ	−0.44	0.45	−1.19
**HDL3 C24:1**	82.1 (17.4)	60.6 (8.3)	144.8 (13.8) ǂ^	−0.56	0.36	−1.30
**HDL3 C26:0**	2.0 (0.3)	1.3 (0.2) ^	1.5 (0.2)	0.36	0.55	−0.22
**VLDL C16:0**	84 (22.4)	119.6 (19.7)	68.9 (17.3)	0.13	−0.28	0.45
**VLDL C18:0**	1.4 (0.4)	1.6 (0.4)	1.3 (0.3)	0.06	−0.08	0.15
**VLDL C20:0**	1.3 (0.4)	1.8 (0.3)	1.4 (0.3)	−0.05	−0.27	0.24
**VLDL C22:0**	14.4 (3.4)	22.6 (3.7)	17.5 (4.0)	−0.15	−0.39	0.22
**VLDL C24:0**	18.9 (4.9)	28.1 (4.2)	22.6 (5.4)	−0.13	−0.33	0.19
**VLDL C24:1**	22.7 (5.5)	33.2 (4.9)	29.7 (6.7)	−0.20	−0.34	0.10
**VLDL C26:0**	0.2 (0.1)	0.3 (0.1)	0.3 (0.1)	−0.23	−0.40	0.19
**HDL2 C16:0**	248 (41.9)	143.9 (21.4) ^	639.7 (91.1) ǂ**	−1.07	0.62	−1.77
**HDL2C18:0**	8 (1.6)	5.9 (0.9)	21.3 (2.7) ǂ**	−1.05	0.32	−1.62
**HDL2 C20:0**	11.4 (2.2)	7.4 (1.2)	31.8 (4.1) ǂ**	−1.14	0.45	−1.76
**HDL2 C22:0**	91.9 (16.9)	70.9 (8.8)	208.9 (27.5) ǂ**	−0.92	0.30	−1.46
**HDL2 C24:0**	190.4 (33.4)	117 (15.2) ^	394.5 (47.1) ǂ**	−0.87	0.57	−1.69
**HDL2 C24:1**	130.6 (23.3)	85.8 (11.9)	317.7 (36.5) ǂ**	−1.07	0.48	−1.79
**HDL2 C26:0**	2.7 (0.4)	1.3 (0.2) *	4.2 (0.6) ǂ^	−0.52	0.81	−1.34

^ *p* < 0.05, * *p* < 0.01; ** *p* < 0.001 compared to Control Group. # *p* < 0.05, ƚ *p* < 0.01, ǂ *p* < 0.001 Compared to Diabetes Macroalbuminuria Group. Data are depicted as Geometric means and associated standard errors. Significance is determined utilizing linear regression models. Overall regression f-test were used to determine species significance and pairwise contrasts used to compare diabetes groups to controls. Between-group effect sizes are presented as Cohen’s d; calculated using mean difference in log_10_ transformed data and pooled standard deviations.

**Table 6:** Lipoprotein levels of sphingoid bases and their phosphates (pmol/mL plasma).

	Group			Cohen’s d		
	Control	D-MA	ND-CKD	Control vs. ND-CKD	Control vs. D-MA	D-MA vs. ND-CKD
**LDL**						
Sphingosine	2.9 (0.5)	4.0 (0.6)	6.2 (1.1) *	−0.74	−0.31	−0.46
Sphingosine 1-phosphate	15 (2.5)	15 (1.8)	19.6 (2.8)	−0.31	−0.00	−0.35
Dihydrosphingosine	1.4 (0.2)	1.7 (0.3)	2.7 (0.5) *	−0.71	−0.22	−0.44
Dihydrosphingosine 1-phosphate	4.9 (0.7)	4.4 (0.5)	6.7 (1.0)	−0.36	0.15	−0.55
**HDL3**						
Sphingosine	9.1 (1.7)	4.8 (0.6) *	3.7 (0.6) **	0.91	0.73	0.30
Sphingosine 1-phosphate	139 (20)	71.8 (9.2) **	108.3 (11.0) #	0.40	0.91	−0.62
Dihydrosphingosine	4.4 (0.7)	2.4 (0.3) *	3.4 (0.5)	0.30	0.74	−0.46
Dihydrosphingosine 1-phosphate	5.9 (1.1)	2.5 (0.4) **	4.5 (0.6) ƚ	0.35	0.95	−0.72
**VLDL**						
Sphingosine	0.6 (0.1)	0.8 (0.1)	1.1 (0.2) ^	−0.51	−0.33	−0.23
Sphingosine 1-phosphate	6.2 (1.3)	7.2 (1.2)	7.0 (1.5)	−0.10	−0.14	0.02
Dihydrosphingosine	0.3 (0.1)	0.3 (0.1)	0.5 (0.1) ^	−0.56	−0.12	−0.45
Dihydrosphingosine 1-phosphate	1.4 (0.3)	1.8 (0.3)	1.7 (0.4)	−0.15	−0.21	0.04
**HDL2**						
Sphingosine	4.7 (0.9)	2 (0.3) **	1.7 (0.3) **	1.07	0.97	0.24
Sphingosine 1-phosphate	14.4 (2.2)	8.3 (1) *	10.4 (1.2)	0.42	0.74	−0.34
Dihydrosphingosine	1.9 (0.3)	1.1 (0.1) ^	1.4 (0.2)	0.37	0.66	−0.30
Dihydrosphingosine 1-phosphate	2.1 (0.3)	1 (0.1) **	1.5 (0.2) #	0.43	0.92	−0.52

^ *p* < 0.05, * *p* < 0.01, ** *p* < 0.001 compared to Control. # *p* < 0.05, ƚ *p* < 0.01, ǂ *p* < 0.001 compared to Diabetes Macroalbuminuria. Data are depicted as geometric means and associated standard errors. Significance is determined utilizing linear regression models. Overall regression f-tests were used to determine species significance and pairwise contrasts were used to compare diabetes groups to controls. Between-group effect sizes are presented as Cohen’s d; calculated using mean difference in log_10_ transformed data and pooled standard deviations.

**Table 7:** Plasma sphingolipid species levels (pmol/mL plasma).

	Group			Cohen’s d		
	Control	D-MA	ND-CKD	Control vs. ND-CKD	Control vs. D-MA	D-MA vs. ND-CKD
**Ceramide**						
C16:0	224.7 (21.2)	220.1 (25.5)	199.5 (35.2)	0.14	0.03	0.11
C18:0	131.3 (9.4)	189.5 (20.9) *	212.1 (16.8) **	−1.04	−0.67	−0.20
C20:0	275.3 (32.7)	377.2 (55.8)	412.2 (53.9) ^	−0.53	−0.39	−0.11
C22:0	597.4 (46.4)	616.4 (56.6)	608.1 (56.4)	−0.03	−0.06	0.03
C24:0	2577.5 (190.1)	2073.1 (219.2)	2221.2 (214.4)	0.29	0.40	−0.12
C24:1	1151.2 (114.9)	983 (85.1)	1056.7 (137.6)	0.12	0.27	−0.11
C26:0	70.6 (6.7)	39.8 (4.7) **	52.2 (6.6)	0.45	0.89	−0.38
**Sphingomyelin**						
C16:0	110,954.4 (5530.4)	102,594.9 (6789.7)	128,419.6 (8693.1) #	−0.41	0.23	−0.57
C18:0	10,349 (301)	10,658.3 (344.5)	10,713.8 (285.7)	−0.20	−0.16	−0.03
C20:0	6021.7 (203.1)	6177.2 (282)	6022.3 (165.5)	−0.00	−0.11	0.12
C22:0	13,880.5 (575.4)	13,961.9 (831.6)	13,731.2 (694.2)	0.04	−0.02	0.05
C24:0	10,665.8 (348.4)	10,250.5 (484.6)	10,452.6 (403.6)	0.09	0.17	−0.08
C24:1	29,946.5 (1228.1)	28,691.8 (1212.5)	30,669.2 (1237.2)	−0.10	0.17	−0.28
C26:0	53 (1.8)	50.7 (2.6)	52.4 (2.1)	0.05	0.18	−0.12
**Lactosylceramide**						
C16:0	1933.5 (172.1)	1848.1 (164.5)	1906.7 (193.8)	0.02	0.08	−0.06
C18:0	88 (11.5)	97.7 (9.7)	66.5 (9.2)	0.34	−0.14	0.54
C20:0	11.2 (1.5)	11 (1.3)	13.2 (1.4)	−0.21	0.02	−0.28
C22:0	59.9 (6.3)	55.2 (5.5)	40.5 (6.4) ^	0.49	0.13	0.40
C24:0	101.5 (8.2)	99.5 (7.2)	77.7 (7.0) #^	0.51	0.04	0.51
C24:1	240.1 (35.5)	188.6 (26.6)	214.2 (31.1)	0.13	0.27	−0.15
C26:0	1.4 (0.3)	2.1 (0.3)	1.1 (0.3) #	0.14	−0.37	0.51
**Hexosylceramide**						
C16:0	1113.9 (102.4)	1224.3 (109.2)	3292.7 (393.1) ǂ**	−1.69	−0.17	−1.59
C18:0	19.3 (2.3)	26.4 (5.4)	53.2 (14.5) #*	−0.82	−0.32	−0.49
C20:0	44.4 (3.6)	47 (4)	100.4 (18.1) ǂ**	−1.00	−0.11	−0.91
C22:0	413.8 (33)	493.2 (37.9)	951.3 (118.7) ǂ**	−1.33	−0.37	−1.07
C24:0	780.4 (64)	712.4 (51.1)	1711.1 (218.0) ǂ**	−1.23	0.19	−1.43
C24:1	690.5 (74.1)	664.8 (50.4)	1675.1 (177.0) ǂ**	−1.35	0.07	−1.70
C26:0	12.2 (1)	9.3 (0.7)	13.8 (1.3) ƚ	−0.22	0.54	−0.76
**Sphingoid bases and their phosphates**						
Sphingosine	27.6 (2.2)	15.0 (1.7) **	16.7 (1.0) **	1.13	1.04	−0.20
Sphingosine 1-phosphate	707.4 (28.6)	610.7 (22.6) ^	596.4 (32.3)^	0.58	0.60	0.09
Dihydrosphingosine	16.6 (1.7)	9.0 (1.2) **	20.5 (2.8) ǂ	−0.29	0.84	−1.03
Dihydrosphingosine 1-phosphate	172.2 (10.5)	149.8 (6.7)	137.9 (12.0)^	0.49	0.42	0.20

^ *p* < 0.05, * *p* < 0.01, ** *p* < 0.001 compared to Control. # *p* < 0.05, ƚ *p* < 0.01, ǂ *p* < 0.001 compared to Diabetes Macroalbuminuria. Data are noted at geometric means and associated standard errors. Significance is determined utilizing linear regression models. Overall regression f-tests were used to determine species significance and pairwise contrasts were used to compare diabetes groups to controls. Between-group effect sizes are presented as Cohen’s d; calculated using mean difference in log_10_ transformed data and pooled standard deviations.

## Data Availability

The raw data supporting the conclusions of this article will be made available by the authors on request.

## Abbreviations

ABCA1	ATP-binding cassette family A protein1
ABCC10	ATP-binding cassette family C protein10
AER	Albumin excretion rate
Apo A1	Apolipoprotein A1
Apo B	Apolipoprotein B
Cer	Ceramides
CKD	Chronic kidney disease
CVD	Cardiovascular disease
GFR	Glomerular filtration rate
GCS	Glucosylceramide synthase
Hex-Cer	Hexosylceramides
HDL	High-density lipoproteins
IDL	Intermediate-density lipoproteins
Lact-Cer	Lactosylceramides
LDL	Low-density lipoproteins
MTP	Microsomal triglyceride transfer protein
VLDL	Very low-density lipoproteins
SM	Sphingomyelin
S1P	Sphingosine 1-phosphate
